# Interactive Dissemination: Engaging Stakeholders in the Use of Aggregated Quality Improvement Data for System-Wide Change in Australian Indigenous Primary Health Care

**DOI:** 10.3389/fpubh.2016.00084

**Published:** 2016-05-03

**Authors:** Alison Laycock, Jodie Bailie, Veronica Matthews, Ross Bailie

**Affiliations:** ^1^Menzies School of Health Research, Charles Darwin University, Casuarina, NT, Australia

**Keywords:** dissemination, knowledge translation, stakeholder engagement, quality, quality of care, primary health care, Indigenous, improvement

## Abstract

**Background:**

Integrating theory when developing complex quality improvement interventions can help to explain clinical and organizational behavior, inform strategy selection, and understand effects. This paper describes a theory-informed interactive dissemination strategy. Using aggregated quality improvement data, the strategy seeks to engage stakeholders in wide-scale data interpretation and knowledge sharing focused on achieving wide-scale improvement in primary health-care quality.

**Methods:**

An iterative process involving diverse stakeholders in Australian Aboriginal and Torres Strait Islander health-care delivery uses aggregated audit data collected across key areas of care. Phases of reporting and online feedback are used to identify: (1) priority areas for improvement; (2) health center, system, and staff attributes that may be important in addressing the identified priority evidence-practice gaps; and (3) strategies that could be introduced or strengthened to enable improvement. A developmental evaluation is being used to refine engagement processes and reports as the project progresses.

**Discussion:**

This innovative dissemination approach is being used to encourage wide-scale interpretation and use of service performance data by policy-makers, managers, and other stakeholders, and to document knowledge about how to address barriers to achieving change. Through the developmental evaluation, the project provides opportunities to learn about stakeholders’ needs in relation to the way data and findings are described and distributed, and elements of the dissemination strategy and report design that impact on the useability and uptake of findings.

**Conclusion:**

The project can contribute to knowledge about how to facilitate interactive wide-scale dissemination and about using data to co-produce knowledge to improve health-care quality.

## Background

Integrating theory when developing and evaluating complex quality improvement interventions can help to explain clinical and organizational behavior, inform strategy selection, and understand effects – thereby developing generalizable knowledge (1, 2), shortening the time needed to identify conditions required for success and optimizing intervention design (3). Researchers and practitioners need to make explicit the theories used (3, 4), as specifying the logic behind continuous quality improvement (CQI) research and practices assists replication and adaptation (5).

Implementation research suggests that by using evidence to identify and link priority gaps in care to theoretical domains that are known to be system enablers or barriers, strategies can be developed that will most likely produce the desired change (6–8). Improvement strategies are more likely to succeed if barriers to effectiveness are identified and addressed at the outset (9, 10).

Despite this evidence, there are few examples of how to select and apply theory when developing implementation interventions (11), and limited examples in the literature of how to choose strategies to overcome barriers to implementing care guidelines (12). This paper describes a wide-scale knowledge translation strategy that draws on implementation theory on addressing barriers to improving health care, to implement what we have termed “interactive dissemination.”

Dissemination is often linked to implementation of research findings, where interventions aim to reduce or remove barriers and promote change – Hailey and colleagues highlight the challenges of matching research findings to the wider perspectives or requirements of groups being addressed (13). Our “interactive dissemination” strategy is consistent with definitions of dissemination as knowledge transfer and exchange, in which there is interactive exchange between researchers and those they intend to influence and an intention to provide and use information as input to decisions or policies leading to change (13–16). The strategy design synthesizes and translates evidence relevant to the CQI program, supports understanding and use of data, and draws on practical knowledge to identify strategies aligned with implementation settings. These elements are identified as necessary for bridging the “how to” gap between dissemination of evidence and implementation in practice (17, 18). Our interactive dissemination strategy, thus, contributes to co-production of knowledge (19, 20), which is inherent in our CQI approach within a national research partnership (21, 22).

### Collaborative Knowledge Production and CQI

There is recognition of the value of collaborative knowledge production processes through which researchers and service providers share explicit and tacit knowledge to find practical, contextually relevant strategies to improve care quality and health outcomes (23, 24). Such processes have potential to help bridge the enduring gap between recommended practice and care delivered (23, 25, 26). Gaps in care provision that occur across multiple health centers are likely to be due to inadequacies in the broader primary health-care (PHC) delivery system. Improving care quality requires change in approaches that operate at multiple levels of the health system and recognize their interdependencies (27). Stakeholders working at different system levels can help in identifying and addressing inadequacies, sharing knowledge to strengthen systems to achieve wide-scale improvement in care delivery, thereby reducing inequities in health-care access and outcomes between population groups (21, 28, 29).

Continuous quality improvement activities are widely implemented in PHC. Typically characterized by feedback of systematically collected data, adaptation to local conditions and involvement of participant leaders, they use iterative processes and recognized change methods (e.g., Six-Sigma, Plan-Do-Study-Act cycles) (30). The participatory nature of CQI enables teams to draw on context-specific and experiential knowledge to develop improvement strategies. There is limited understanding of how these CQI principles and processes can be applied at scale to achieve system-wide improvement.

### An Interactive Dissemination Strategy Using Aggregated CQI Data

The dissemination strategy uses CQI data from a program of CQI research and development in Aboriginal and Torres Strait Islander (Australia’s Indigenous peoples) PHC in Australia (Box 1). Data contributed over 8 years by 175 health centers delivering care to Indigenous people are aggregated at the national level, and at the Australian state/territory level where sufficient data are available. They comprise clinical audit data on adherence to best practice guidelines representing 56,000 patient records, and data from 492 systems assessments completed by health teams, in priority aspects of PHC. Evidence on this scale enables identification of gaps in care that occur across health centers, and offers a foundation for developing evidence-informed policies and programs to achieve high-level system change and large-scale improvement.

Box 1 **The Audit and Best Practice for Chronic Disease (ABCD) National Research Partnership.**

In 2010, the ABCD National Research Partnership brought together PHC services, policy, and support organizations and research institutions to guide and support research in improving the quality of Indigenous PHC across Australia (22). Concurrently, the National Centre for Quality Improvement in Indigenous Primary Health Care (www.One21seventy.org.au) was established to provide tools, processes and training to support CQI and strengthen the implementation of clinical care guidelines. Almost 80% of health centers using One21seventy services agreed to share their de-identified CQI data for research purposes, forming the most comprehensive and broad-scale dataset relating to health center performance currently available for Indigenous PHC.

Partnership research has highlighted wide variation in performance between different aspects of care and between health centers. While many aspects of care are delivered well in many health centers, there are important gaps between evidence and practice in some aspects of PHC (31–33).

### Engaging Stakeholders in the “Identifying Priority Evidence-Practice Gaps, Barriers and Strategies for Improvement (ESP)” Project

The purpose of the interactive dissemination strategy – the ESP project – is to engage stakeholders working in Indigenous PHC delivery, management, policy, and research with these aggregated CQI data in order to:

- obtain input in identifying priority evidence-practice gaps, barriers and enablers to addressing the identified priority evidence-practice gaps, and strategies for improvement, and
- encourage use of the data and findings for policy and program development and systems change.

Targeted stakeholders include health practitioners (e.g., doctors, nurses, allied health professionals, Indigenous Health Practitioners), managers and policy-makers working at various levels of the health system, researchers, staff of health service support organizations, and peak bodies representing the interests of Indigenous communities and community-controlled health services.

### Context

Indigenous Australians experience an inequitable burden of ill-health, shorter life expectancy and poorer access to health services compared with the general population (34, 35). Contributing factors are complex, relating to colonization and discrimination, social and economic inequalities, and cultural safety. Indigenous Australians access PHC through Indigenous community-controlled health and government-managed services designed to meet their needs (36), and through private general practices. Indigenous PHC settings are diverse in geography, governance, and resource provision, and characterized by complex political, cultural, and social interactions.

Continuous quality improvement activities are implemented in many PHC centers that serve Indigenous people, for example, through use of audit and system assessment tools, and Plan-Do-Study-Act approaches. A national CQI framework for Aboriginal and Torres Strait Islander PHC (37) is being established. In this complex health-care environment, it is important to build on strengths and existing knowledge, making optimal use of CQI data and research to help address health inequities.

## Methods

### Theoretical Framework

The ESP project design is adapted from systematic methods designed to link interventions to modifiable barriers to address evidence-practice gaps. French and colleagues designed a four-step process comprising guiding questions to identify: (1) an evidence-practice gap, and what needs to be done differently by whom to reduce it; (2) barriers that should be addressed by intervention strategies, based on previously tested theoretical domains relevant to behavior change of health-care professionals (7, 8); (3) intervention components that could overcome the barriers and enhance enablers, and; (4) how behavior change can be measured and understood (6). French et al.’s process has provided the theoretical base for the design of the ESP project, which is guided by the questions: “What are the priority evidence-practice gaps evident in the aggregated CQI data?” “Which barriers and enablers need to be addressed?” “Which strategies could overcome modifiable barriers and enhance enablers?” and “How can we improve dissemination methods to encourage engagement with the data and use of findings?”

### Iterative Participatory Approach

The ESP project uses an iterative and participatory approach. Drawing on action research principles, cycles of systematic enquiry, collaboration, and refinement are applied for the purpose of effecting change (38) and developing theoretical understanding (39).

Separate ESP processes are implemented using audit data collected for child health, chronic illness care, rheumatic heart disease, preventive, maternal, and mental health care. Each process comprises four phases of reporting and stakeholder feedback, culminating in a final report. Each phase comprises a report and linked online survey that uses Likert-scale and open-ended questions to elicit interpretive and reflective responses. We distribute the reports by email to people in partner organizations and extended networks, encouraging further distribution, discussion, and facilitated group input. The survey tool distinguishes between individual and group responses.

#### Phase 1

The first report includes the most recent available CQI data in one aspect of PHC delivery (e.g., child health), aggregated and presented as box and whisker plots with interpretive information and preliminary analysis. This analysis is done by the research team, in collaboration with clinical experts, to identify priorities for improvement. Through the phase 1 survey, we seek feedback on the preliminary priorities, whether they align with respondents’ pre-existing perspectives on priorities for improvement and whether other priorities should be included.

#### Phase 2

The second report includes the findings from the phase 1 survey (consensus evidence-practice gaps) and trend data over time and by audit cycle for indicators relevant to the identified improvement priorities. We ask respondents to reflect on the trend data and their experience, and answer survey questions to rate potential barriers to improving the priority gaps experienced at different levels of the health system, including system factors relevant to the Indigenous PHC sector (40). Listed domains relating to health center systems, the broader system environment, and staff attributes are drawn from international and national research (7–9, 40, 41). Questions about barriers and enablers relating to individual attributes are informed by the Theoretical Domains Framework (7, 8, 42, 43). Respondents are also asked to rate the accessibility, usability, and usefulness of the report and suggest improvements.

#### Phase 3

The third report includes the Phase 2 findings and a summary of published evidence about successful strategies used in CQI, which is intended to stimulate thought and discussion about possible strategies for improving care. We use the Phase 3 survey to find out how stakeholders think existing strategies could be refined, or new strategies developed, to build on system strengths and enablers and overcome the main barriers to addressing the priority evidence-practice gaps. Respondents are also asked if the report provides a fair reflection of the main barriers and enablers to improvement in relation to the priority evidence-practice gaps, and how the report could be refined.

#### Review

The team incorporates feedback to develop and distribute a draft report of the overall project findings and invites project participants to comment on the representation of findings using a brief online survey. Responses are used to finalize the ESP report in that particular aspect of PHC.

**Figure 1:**
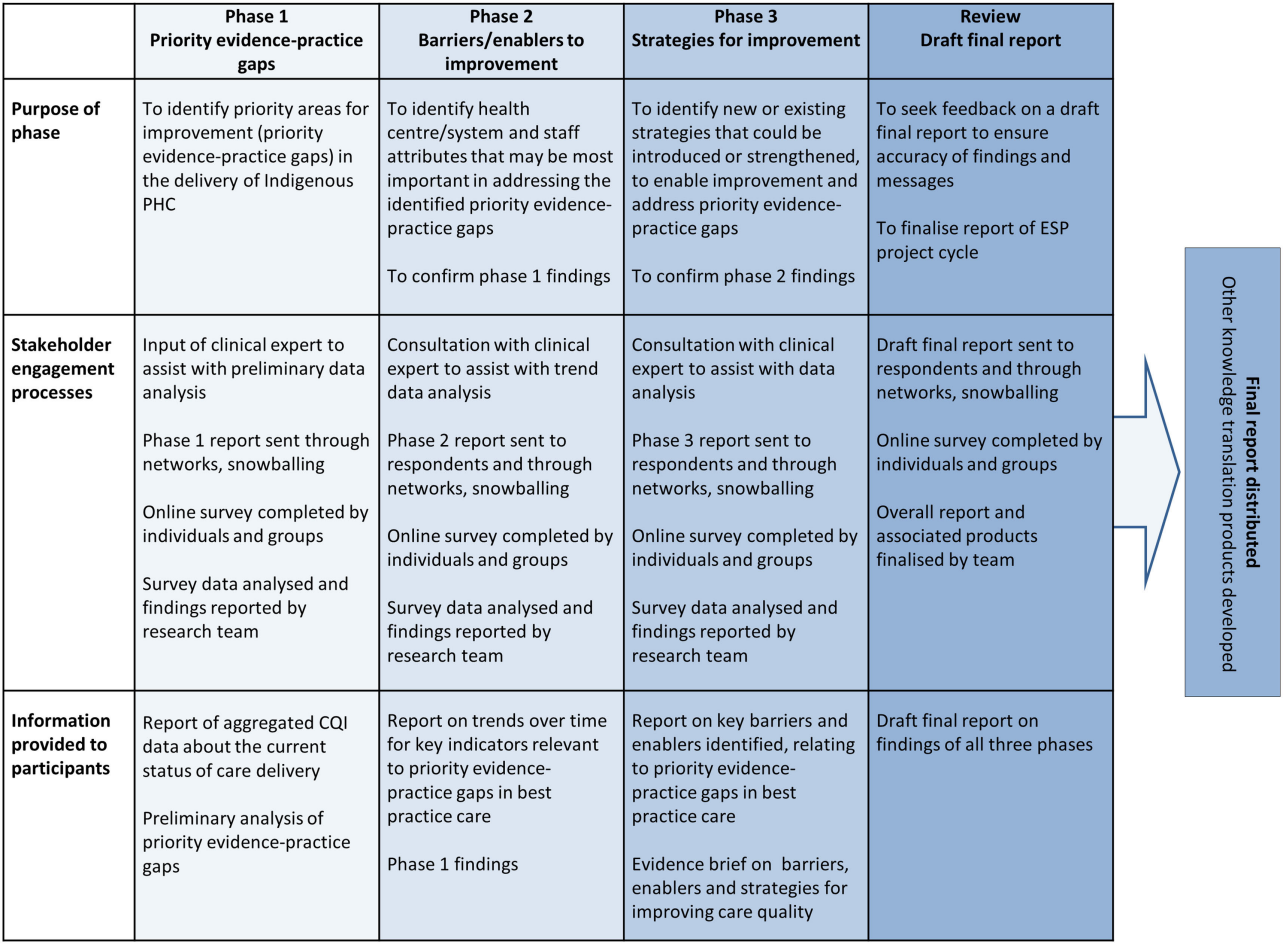
**Phases of the ESP project**. Note: this process is repeated for each area of care (e.g., child health, chronic illness care). Source: Matthews et al. (26). PHC, primary health care; CQI, continuous quality improvement; ESP, “Engaging Stakeholders in Identifying Priority Evidence-Practice Gaps, Barriers and Strategies for Improvement (ESP)” Project.

The purpose and elements of ESP phases are summarized in Figure 1.

### Concurrent Developmental Evaluation

A developmental evaluation is being conducted to refine the ESP project structure, materials, and processes as it progresses. One member of our research team (AL) has the lead role on the evaluation, which is expected to contribute to the team’s learning and the project’s dissemination goals. The evaluation method and resulting project modifications will be described separately.

## Discussion

### Use of Aggregated Data for Wide-Scale Quality Improvement

There is need for innovative dissemination approaches that encourage use of service performance data by policy-makers, managers, practitioners, and community members to identify and address barriers to achieving change. Researchers need to be involved in dialog with these groups to understand policy contexts and how evidence may translate into action (44), and to plausibly link the development of scientifically sound advice with knowledge exchange processes (45). A recent systematic review found timely access to good quality relevant research evidence, collaborations, relationship- and skills-building to be important factors influencing policy-makers’ use of evidence (46). An Australian review found limited evidence that managers and policy-makers could use to assess the impact of system- and service-level attributes on health outcomes for Indigenous peoples, concluding that more mixed-method research that includes multiple stakeholder perspectives, including those of Indigenous community members, is required (28).

Continuous quality improvement programs typically bring health teams together to plan evidence-informed improvements utilizing clinical data and contextual knowledge to address local evidence-practice gaps in care. In this large-scale project, the challenge of engaging people in “discussion” about care quality based on aggregated data is heightened by limited opportunities for face-to-face or individual-level communication between research team members and stakeholders. Research is needed to determine how CQI processes can be scaled up for higher-level policy and management purposes. It stands to reason that interpretation and use of aggregated CQI data and input by stakeholders in varying roles has potential to identify common and important improvement priorities, and to utilize the collective strengths within PHC services to continue improving health-care quality for Indigenous Australians.

### Opportunities for Learning about What Works in Dissemination and Knowledge Co-Production

Through the developmental evaluation, the team expects to learn more about stakeholders needs and preferences in relation to the way data and findings are described and distributed, and elements of the dissemination strategy and report design that impact on the usability and uptake of findings (47) – including the use of implementation science theory. There is a positive correlation between stakeholder engagement in knowledge production and implementation (23). We hope that by developing understanding of factors that impact on stakeholder participation in the project, and gathering feedback about how to better capture and present stakeholder input, we can contribute knowledge to strengthen the design and impact of knowledge translation processes.

The project should assist in understanding the potential and limitations of online communication to engage health-care stakeholders in wide-scale interactive dissemination processes.

### Opportunities for Learning about What Works to Improve PHC Systems and Quality

The input provided by stakeholders on barriers and enablers, and on strategies for improvement, is valuable in that it reflects tacit knowledge of people working within the health system. We have made innovations to an existing implementation tool used for exploring individual attributes that influence care. Additional questions in the tool are designed to capture knowledge about determinants of performance that operate at health center and system levels (40, 41). This exploratory work may inform further studies in health systems and implementation research, including the development of tools to identify barriers to improvement at multiple system levels.

The CQI process used to assess health center systems includes a domain about community linkages (48). A priority for improvement reflected in the aggregated system assessment data is the strengthening of links between health centers and Indigenous communities. Related enablers identified through the ESP process to date include strengthening of community engagement in service delivery design and community leadership for CQI (26). The ESP process has a higher system focus than the health center CQI process; therefore, input from Indigenous peak bodies is important for achieving linkages to influence policy and program design at higher system levels.

The design of wide-scale improvement strategies in the Australian Indigenous PHC context needs to reflect understanding of the holistic nature of Indigenous wellbeing beyond physical health, including healthy connections to culture, community, and land, as well as published evidence and expert knowledge. Findings relating to identified barriers, enablers, and strategies will be reported separately.

Documentation and evaluation of implemented strategies will contribute knowledge about what works and in what contexts to improve PHC for Indigenous communities, and will support adaptation to other settings.

### Strengths and Limitations

A strength of the ESP project is its iterative design using multiple phases. In conjunction with the developmental evaluation, implementing a new dissemination process with each PHC audit tool dataset provides the team with multiple opportunities to reflect and respond to stakeholder feedback, drawing on evidence and available resources to make and test refinements to processes, reports, and supporting materials. To our knowledge, the level of detail of the data made available from a large number of services across wide geographic scope through this project has not been achieved by other projects.

We are using an open process to engage stakeholders, inviting those who receive reports to distribute them online through their workplaces and networks, and respond to surveys individually or through groups. This strength in the project design puts no limit on the number and diversity of possible participants, thereby enhancing data interpretation and enriching knowledge sharing. We encourage peak bodies representing Indigenous communities to use the reports as a basis for group discussion, enabling further opportunity for community members’ input.

The open process also makes it difficult to assess reach and response rates relative to distribution. On balance, the advantages of this snowballing distribution process outweigh the potential limitation in relation to accurate reporting of survey distribution and responses, as a goal of the project is to provide wide-scale access to these CQI data and ESP project findings.

## Conclusion

The ESP project uses an innovative theory-informed approach to advance the use of large-scale aggregate CQI datasets, enabling a range of stakeholders to identify priority gaps and related barriers in the delivery of best practice PHC in Indigenous communities. Using aggregate CQI data to stimulate discussion among diverse stakeholders on priority evidence-practice gaps in care, and how best to achieve improvement, will contribute knowledge about how to facilitate interactive dissemination and data use.

This process will identify major themes for improving PHC delivery through changes at the health center and community, regional, and national levels. We expect common themes identified across key areas of PHC to be relevant to developing policy and implementing large-scale change to strengthen systems and improve the provision of comprehensive PHC for Indigenous communities across Australia. We anticipate that lessons learned about applying theory to inform the development of improvement interventions, and engaging stakeholders in use of aggregated CQI data for knowledge co-production and system-wide change, will be transferable to other settings.

## Ethics Statement

Ethical approval for the ABCD National Research Partnership was obtained from research ethics committees in each relevant Australian jurisdiction – Human Research Ethics Committee of the Northern Territory Department of Health and Menzies School of Health Research (HREC EC00153), Central Australian Human Research Ethics Committee (HREC-12-53), New South Wales Greater Western Area Health Service Human Research Committee (HREC/11/GWAHS/23), Queensland Human Research Ethics Committee Darling Downs Health Services District (HREC/11/QTDD/47), South Australian Aboriginal Health Research Ethics Committee (04-10-319), Curtin University Human Research Ethics Committee (HR140/2008), Western Australian Country Health Services Research Ethics Committee (2011/27), Western Australia Aboriginal Health Information and Ethics Committee (111-8/05), and University of Western Australia Human Research Ethics Committee (RA/4/1/5051). All participants in the ESP Project surveys and evaluation provide individual informed consent.

## Author Contributions

AL planned and wrote the manuscript. JB provided input and commented on all drafts. VM contributed to conception of the ESP project, led the quantitative analysis of the ABCD data and commented on the draft. RB is the leader of the ABCD National Research Partnership, of which the ESP project is a dissemination strategy. He played a lead role in conceptualization of the ESP process, and contributed to conceptualization and review of the manuscript. All authors read and approved the final manuscript.

## Conflict of Interest Statement

The authors declare that the research was conducted in the absence of any commercial or financial relationships that could be construed as a potential conflict of interest.

## Abbreviations

ABCD, audit and best practice for chronic disease; CQI, continuous quality improvement; ESP, engaging stakeholders in identifying priority evidence-practice gaps, barriers and strategies for improvement; PHC, primary health care.
